# Macrophages are the target cells of genotype VII Newcastle disease virus and promote the infection and apoptosis of chicken splenic T cells

**DOI:** 10.1186/s13567-025-01631-8

**Published:** 2025-10-30

**Authors:** Miao Cai, Qing Chen, Yifei Liu, Cong Wang, Muyao Li, Xiaolong Lu, Tianxing Liao, Xiaowen Liu, Xiaoli Hao, Yu Chen, Shunlin Hu, Xiufan Liu

**Affiliations:** 1 Key Laboratory of Avian Bioproducts Development, College of Veterinary Medicine, Yangzhou University, Yangzhou, 225000 China; 2 Jiangsu Co-Innovation Center for Prevention and Control of Important Animal Infectious Diseases and Zoonosis, Yangzhou University, Yangzhou, 225000 China; 3 Jiangsu Key Laboratory of Zoonosis, Yangzhou University, Yangzhou, 225000 China

**Keywords:** Newcastle disease virus, flow cytometry analysis, lymphocytes, macrophages, T cells, apoptosis

## Abstract

Infection with genotype VII Newcastle disease virus (NDV) poses a substantial threat to poultry health and is characterized by severe damage to immune organs, with effects such as lymphocyte depletion. However, the precise mechanisms underlying this phenomenon remain unclear. Our present investigation focused on the dynamic changes in macrophages, T cells, and B cells in the spleens of chickens infected with genotype VII NDV by using multicolour flow cytometry. We found that NDV selectively infected chicken splenic macrophages and significantly increased the number of macrophages at 4 days post-infection. In contrast, T and B cells became progressively depleted. In vitro experiments revealed that following genotype VII NDV infection, T cells underwent apoptosis more potently when they were cocultured with macrophages than when they were not. Overall, our findings highlight the changes in chicken splenic immune cell populations triggered by genotype VII NDV and illuminate the role of macrophages in T-cell depletion.

**Supplementary Information:**

The online version contains supplementary material available at 10.1186/s13567-025-01631-8.

## Introduction

Newcastle disease (ND) is an acute, highly contagious avian infectious disease caused by Newcastle disease virus (NDV) and has caused pandemics worldwide [1]. NDV, which belongs to the genus Orthoavulavirus, is classified into avirulent (lentogenic), intermediate (mesogenic), and virulent (velogenic) pathotypes based on pathogenicity [2]. Phylogenetic analysis of the fusion (F) gene further categorized NDV into two classes, class I and class II, with genotype VII strains currently driving the fourth and ongoing pandemic [3–5]. Virulent NDV strains are typically lymphophilic and can cause lymphocyte depletion in the spleen, bursa, and thymus, along with significant upregulation of genes involved in the innate immune response [6–8]. NDV viral loads in these organs correlate well with the severity of clinical signs and tissue damage [9]. Compared with other genotype strains, genotype VII NDV can cause more severe damage to the immune organs of poultry, inducing a strong immune response and cell death in lymphoid tissues, particularly in the spleen [10]. Therefore, even if vaccinated with traditional vaccines, immunized poultry flocks can still be infected by genotype VII NDV, leading to a reduction in protective efficacy.

In chickens, splenic mononuclear cells (SMNCs) primarily consist of lymphocytes with a small quantity of macrophages and dendritic cells (DCs) [11–13]. Based on available antibodies specific to chicken immune cell surface markers as previously published [11], we developed a multicolour flow cytometry protocol to precisely quantify splenic macrophages and lymphocytes, enabling detailed analysis of their dynamics post-NDV infection.

Macrophages are essential for maintaining homeostasis in the body and play a central role in the antiviral immune response. Macrophages are innate immune cells that release inflammatory factors during viral invasion. They also function as antigen-presenting cells, contributing to both innate and adaptive immune responses. Additionally, they can engulf pathogens and clear cellular debris through phagocytosis [14, 15]. Their longevity and migratory capacity also make them ideal targets for viral exploitation [16–19]. NDV has an affinity for macrophages and can enter chicken macrophages through pH-dependent, dynamin-mediated endocytic pathways involving small vesicles [20], inducing macrophage apoptosis, subverting host immunity and exacerbating tissue injury [21]. Thus, macrophages may critically influence NDV pathogenicity.

Cell-mediated immunity (CMI), orchestrated by T lymphocytes, is essential for antiviral defence. In the spleen, CD4^+^ T helper cells, CD8^+^ cytotoxic T cells, and γδ T cells drive CMI responses. While αβ T cells recognize antigen‒MHC complexes on antigen-presenting cells (APCs), γδ T cells directly detect pathogens and mediate cytotoxicity [22–24]. CMI responses emerge as early as 2–3 days post-NDV infection or vaccination [25, 26] and likely mitigate viral spread by eliminating infected cells [27].

Macrophages and T lymphocytes are important immune cells in the chicken spleen and play critical roles in viral infection and immunity. However, the dynamic changes in immune cell populations within the chicken spleen during NDV infection, along with the mechanisms driving the significant depletion of T lymphocytes, remain poorly characterized. Here, we analysed the dynamic changes in the main splenic immunocytes after genotype VII NDV infection and investigated the relationships between macrophages and T cells in the opposite direction. Our findings provide insights into the pathogenesis of genotype VII NDV-induced splenic damage.

## Materials and methods

### Ethics statement

This study was carried out in strict accordance with the recommendations in the Guide for the Care and Use of Laboratory Animals of the Ministry of Science and Technology of the People’s Republic of China. All experiments involving NDV were executed in the animal biosafety level 3 facility (CNAS registration No. CNAS BL0015) at Yangzhou University in strict accordance with the recommendations of the institutional biosafety manual and supervised by the Institutional Biosafety Committee of Yangzhou University.

### Cells and virus

Chicken SMNCs were isolated from 4-week-old specific pathogen-free (SPF) white leghorn chickens through density gradient centrifugation using a Chicken Splenic Mononuclear Cell Isolation Kit (Haoyang, Tianjin, China). SMNCs were grown in RPMI 1640 medium supplemented with 10% foetal bovine serum (FBS) (Thermo Fisher Scientific, Waltham, MA, USA) at 37 ℃, and 5% CO_2_. T cells were sorted from SMNCs and cultured in MH-S-specific culture medium (RPMI 1640 medium supplemented with 10% FBS, 0.05 mM β-mercaptoethanol and 1% P/S; Procell, Wuhan, China) at 37 ℃, and 5% CO_2_. The macrophages were also sorted from the chicken SMNCs and cultured in MH-S-specific culture medium at 37 ℃, and 5% CO_2_. The recombinant genotype VII NDV strain rI4-EGFP was generated in our laboratory by inserting an EGFP tag at the C-terminus of the HN protein in the parental strain JS-5-05-Go (GenBank: JN631747.1). This recombinant virus was preserved in our laboratory and propagated in 9-day-old SPF chicken embryos.

### Antibodies and reagents

Mouse anti-chicken Bu-1-AF647 monoclonal antibody (8395-31), mouse anti-chicken CD45-SPRD monoclonal antibody (8270-13), mouse anti-chicken monocyte/macrophage-PE monoclonal antibody (8420-09), mouse anti-chicken CD3-SPRD monoclonal antibody (8200-13), mouse anti-chicken TCRγδ-BIOT monoclonal antibody (8230-08), mouse anti-chicken CD8α-AF700 monoclonal antibody (8220-27) and mouse anti-chicken CD4-PACBLU monoclonal antibody (8210-26) were purchased from Southern Biotech (Birmingham, AL, USA). Brilliant Violet 510 Streptavidin (405233) was purchased from BioLegend (San Diego, CA, USA). CD3 monoclonal antibody (Biotin, MA5-28695) and Fixable Viability Dye eFluor 780 (65-0865-14) were purchased from Thermo Fisher Scientific (Waltham, MA, USA). Antibiotin microbeads (130-090-485) and MS separation columns (130-042-201) were purchased from Miltenyi Biotec (Bergisch Gladbach, Germany).

### Challenge experiments in chickens

Thirty four-week-old SPF chickens were randomly divided into two groups. Fifteen of them were inoculated with the recombinant virus rI4-EGFP via eye drops at a dose of 10^5^ EID_50_, while the other 15 were inoculated with an equal volume of sterile phosphate-buffered saline (PBS) through the same route and served as the control group. On Days 2, 4, and 6 post-infection, five chickens were randomly selected from each group and euthanized. The spleens were then harvested, and an SMNC suspension was prepared for cell phenotype analysis.

### Splenic mononuclear cell preparation

The chicken SMNCs were constructed following the manufacturer’s instructions. The whole spleen was mechanically disrupted, and an appropriate amount of tissue homogenization liquid was added. Then, the mixture was pushed through a 70 µm cell strainer (Corning, NY, USA) using a 5 mL syringe plunger. The cells were then centrifuged at 400 × *g* for 10 min. After the supernatant was discarded, the cells were resuspended in an appropriate amount of sample dilution liquid and layered onto an equal volume of SMNC separation liquid, followed by centrifugation at 500 × *g* for 25 min. The SMNCs were collected and washed. The isolated cells were resuspended in complete RPMI-1640 medium. The final cell concentration was adjusted to 1 × 10^7^ cells/mL. Cell counts were performed using a SmartCell 200 (SC1001) purchased from Monwei (Shanghai, China).

### Cell sorting by flow cytometry

A total of 4 × 10^7^ cells were collected and centrifuged at 300 × *g* for 5 min. After the supernatant was discarded, a 1 mL antibody cocktail containing anti-chicken monocyte/macrophage, Bu-1, and CD3 antibodies was added to each sample tube in the dark at 4 ℃ for 30 min. Control tubes without antibodies and single positive tubes for the three antibodies were also prepared for compensation adjustment. After the cells were washed with flow buffer, 1 mL of buffer was added to each tube to resuspend the cells. A FACSAria SORP (Becton Dickinson, USA) was used to sort the various cells, and a collection of 1–2 × 10^6^ cells was obtained.

### Chicken splenic T-cell sorting by magnetic beads

A total of 10^8^ cells were collected and centrifuged at 300 × *g* for 5 min. Then, the supernatant was discarded, and the cells were resuspended in 1 mL of MACS buffer. After centrifugation, the cells were resuspended in 1 mL of MACS buffer. Then, 13 μL of CD3-biotin antibody was added, and the mixture was incubated at 4 ℃ for 30 min. After centrifugation, the cells were washed once with MACS buffer and resuspended in 475 μL of MACS buffer. Twenty-five microlitres of magnetic beads was added, mixed well, and incubated at 4 ℃ for 15 min. After centrifugation and washing, the cells were resuspended in 500 μL of MACS buffer, and sorting was performed using a magnetic rack. The sorted T cells were cultured in MH-S medium at 37 ℃ with 5% CO_2_.

### Purification of chicken splenic macrophages

In reference to the separation of macrophages from peripheral blood mononuclear cells (PBMCs) [28], a two-step adherence culture method was used to separate macrophages from splenic cells. Chicken SMNCs were cultured at a density of 1 × 10^7^ cells per well in a 6-well plate. At 24 h and 48 h post-culture, the cells were washed twice with PBS to remove nonadherent cells. The remaining adherent cells were used for subsequent experiments.

### Quantitative real-time RT‒PCR (qRT‒PCR) analysis of viral load

Viral mRNA was extracted from the cells using the Universal RNA Extraction Kit (2161; GENENODE, China) after infection with rI4-EGFP. Afterwards, cDNA synthesis was performed using HiScript II Q Select RT SuperMix for qPCR (+gDNA wiper) (R233-01; Vazyme, Nanjing, China), and qRT‒PCR was performed using AceQ qPCR Probe Master Mix (Q112-02; Vazyme, Nanjing, China) according to the manufacturer’s instructions on a LightCycler 480 (Roche, Basel, Switzerland). The specific primers and probes used for qPCR are listed in Table 1.

**Table 1:** **Primers and probes for qPCR of the F gene**

Primer	Sequence (5′–3′)
I4-F	GGTCAATCATAGTCAAGTTGCTCC
I4-R	AACCCCAAGAGCTACACTGCC
Probe	FAM-AAGCGTTTTTGTCTCCTTCCTCC-BHQ1

### Establishment of the macrophage–T-cell coculture model and Transwell coculture assay

We established a macrophage–T-cell coculture model on the basis of a previous study [29]. The macrophages were detached with trypsin, counted, seeded at 1 × 10^6^ per well in a 12-well plate, and cultured overnight in RPMI 1640 medium supplemented with 10% FBS and 1% penicillin/streptomycin. Subsequently, the medium was carefully removed, and 4 × 10^6^ sorted T cells were added to each well, followed by the addition of fresh MHS-specific medium for continued culture. Transwell inserts (Costar, Cambridge, MA, USA) with a 0.4 µm pore membrane were used to separate the upper and lower compartments. This prevented direct cell contact while permitting the diffusion of soluble factors. Macrophages were seeded at a density of 1 × 10^6^ cells in the upper chamber, and purified T cells were seeded at a density of 2 × 10^6^ cells in the lower chamber.

### NDV infection of SMNCs, T cells, and cocultured cells

SMNCs and sorted T cells were seeded at 3 × 10^6^ cells per well in a 12-well plate, and cocultured cells were seeded at 5 × 10^6^ cells per well in a 12-well plate. These cells were infected with the rI4-EGFP strain at the indicated multiplicity of infection (MOI) for a designated period and were subsequently cultured in MHS-specific medium at 37 °C under a 5% CO_2_ atmosphere. After viral infection, the cell samples were used for subsequent analysis.

### Analysis of different cell population percentages and apoptosis rates by flow cytometry

Three flow cytometry staining panels were designed on the basis of a previous study [11], with Panel 1 for detecting macrophages and B cells and myeloid lineage, Panel 2 for detecting T-cell subsets, Panel 3 for assessing T-cell viability, and Panel 4 for assessing the apoptosis of macrophage and T-cell subsets. The specific protocols are shown in Table 2, and the gating strategies are shown in Additional files 1 and 2. One hundred microlitres of cell suspension, adjusted to a concentration of 2 × 10^6^ cells, was added to 1.5 mL tubes for antibody staining and final flow analysis. The cells were subsequently centrifuged at 300 × *g* for 5 min, after which the supernatant was discarded. The cells were resuspended in FACS buffer (0.5% BSA in PBS from Sigma), and anti-chicken antibodies were added. The mixture was incubated at 4 °C in the dark for 30 min. After centrifugation, the cells were resuspended in 1 × binding buffer, and 5 μL of Annexin V was added (88-8103-72; Thermo Fisher Scientific, Waltham, MA, USA). The mixture was subsequently incubated in the dark at room temperature for 10 min. The cells were then washed twice with flow cytometry buffer and finally resuspended in 400 μL of the same buffer for FACS analysis. Concurrent with surface staining in the experimental wells, blank control wells (without any antibodies) and single positive control wells for all antibodies (for compensation adjustment) were also established. The data were analysed with FlowJo software (Tree Star Inc., USA).

**Table 2:** **The multicolour flow cytometry panel designed for SMNC analysis**

Fluorochrome	APC	APC-780	PE	PerCP-Cy5.5	Pacific Blue	BV510	Alexa eFluor 700	PE-cy7
Panel-1	Bu-1		KUL01	CD45				
Panel-2				CD3	CD4	TCR1	CD8ɑ	
Panel-3		FVD		CD3				
Panel-4			KUL01	CD45	CD4		CD8ɑ	Annexin-V

### Western blot analysis of viral protein expression

SMNCs and T cells were treated as indicated and then washed three times with cold PBS before being lysed in RIPA buffer (P0013B; Beyotime Biotech, Shanghai, China) supplemented with the proteinase inhibitor PMSF (ST506; Beyotime Biotech, Shanghai, China). The total protein concentration in the cell lysate was then measured using a BCA protein assay kit (P0012; Beyotime Biotech, Shanghai, China). The denatured proteins were separated using 10% SDS‒PAGE and further transferred to polyvinylidene difluoride (PVDF) membranes. The PVDF membranes were subsequently blocked and incubated with diluted primary and secondary antibodies. Detection was performed by incubating the membranes with a chemiluminescent substrate and exposing them in a dark room with a ChemiDoc Imager (Bio-Rad Laboratories, CA, USA).

### Analysis of the apoptosis of macrophages, T cells, and cocultured cells

The apoptosis ratio was measured with an Annexin V-FITC/PI apoptosis detection kit (C1062M; Beyotime Biotech, Shanghai, China) according to the manufacturer’s instructions. Briefly, the macrophages were trypsinized with non-EDTA-digested trypsin, and the T cells were collected by centrifugation at 500 *g* and 4 °C for 5 min. Then, the cells were washed three times with PBS and resuspended in 195 μL of prechilled Annexin V-FITC binding buffer supplemented with 5 μL of Annexin V-FITC and 10 μL of PI. The cells were incubated at room temperature for 10 min in the dark. After incubation, 400 μL of Annexin-binding buffer was added, and the samples were immediately analysed by a FACS LSRFortessa (BD Biosciences, Franklin Lakes, NJ, USA).

### Statistical analysis

All the data are presented as the means ± SDs as indicated. Student’s *t* test and one-way and two-way ANOVA were used for the analysis of studies where appropriate. All the statistical analyses and calculations were carried out using GraphPad Prism software (San Diego, USA). A *P* value of less than 0.05 was considered to indicate statistical significance. NS indicates no significant difference; *P < 0.05, **P < 0.01, ***P < 0.001, ****P < 0.0001.

## Results

### Dynamic changes in SMNCs following infection with genotype VII NDV

**Figure 1:**
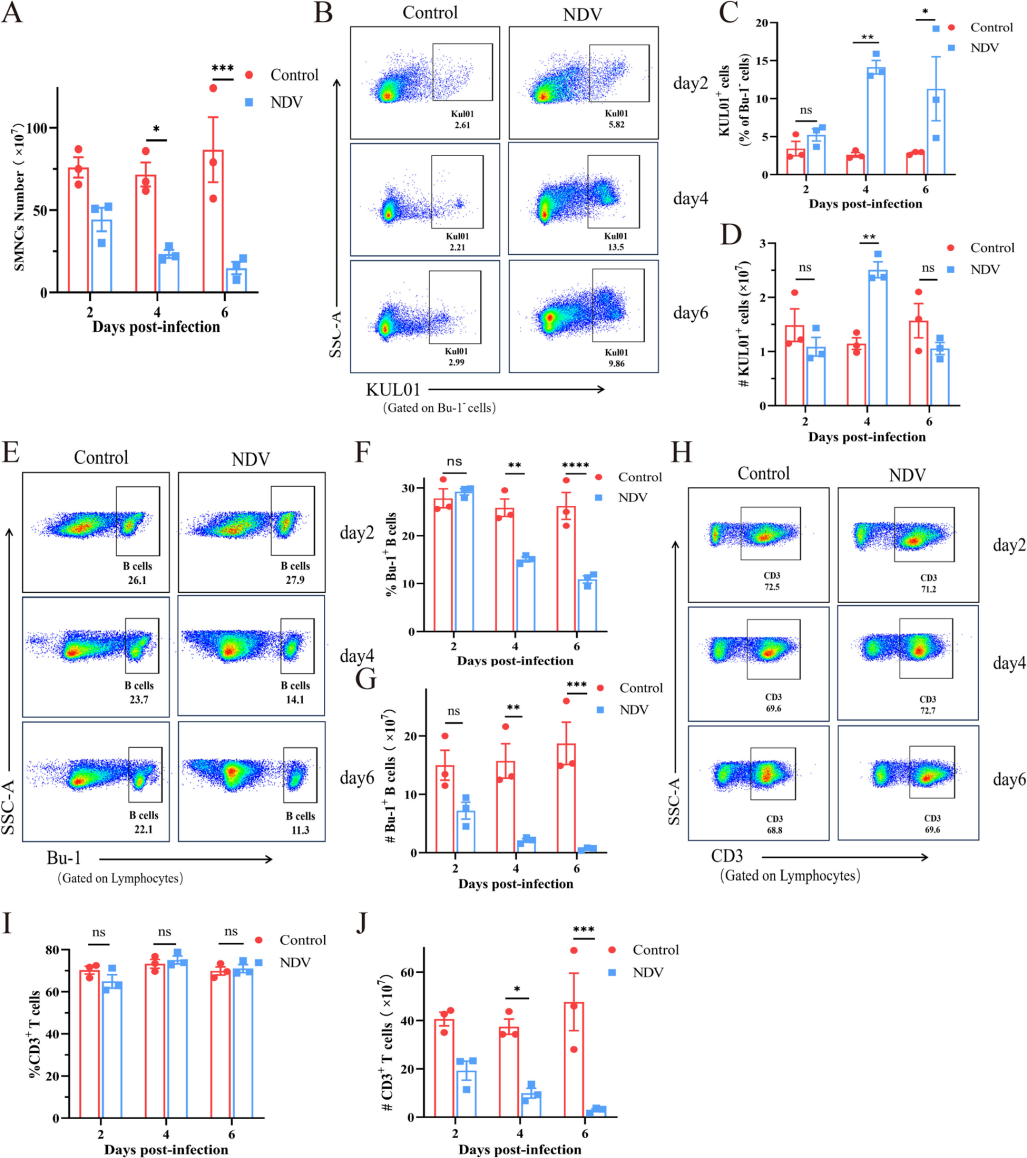
**Dynamic changes in SMNCs after infection. **(**A)** The total number of SMNCs in the entire spleen of chickens was determined in both the control and infected groups at 2, 4, and 6 dpi. (**B**) Representative dot plots depicting KUL01 + cells in the spleens of chickens. The percentages (**C**) and numbers (**D**) of KUL01^+^ cells in the spleens were measured. (**E**) Representative dot plots showing the number of Bu-1^+^ B cells and the percentages (**F**) and total numbers (**G**) of Bu-1^+^ B cells in the spleen. (**H**) Representative dot plots depicting CD3^+^ T cells in the spleens of chickens**.** The percentages (**I**) and total numbers (**J**) of CD3^+^ T cells in the spleen were also measured.

To elucidate the dynamics of immune cells in the spleen of chickens following genotype VII NDV infection, we conducted a comprehensive analysis of splenocyte populations. Spleens from infected chickens were harvested, and single-cell suspensions were prepared and counted for detailed immunophenotyping using flow cytometry with specific markers for cell labelling. Our data revealed a significant decrease in the total number of SMNCs over time post-infection, with the most pronounced decline occurring at 6 dpi (Figure 1A). The proportion of macrophages in the infected group did not significantly differ from that in the control group at 2 dpi but significantly increased at 4 dpi and 6 dpi (Figures 1B and C). The absolute number of macrophages also significantly increased at 4 dpi (Figure 1D). In terms of B cells, the proportion in the infected group did not significantly differ from that in the control group at 2 dpi but significantly decreased afterwards, with a sharp decrease at 6 dpi (Figures 1E and F). The number of B cells in the infected group was consistently lower than that in the control group, with a sharp decrease at 6 dpi (Figure 1G). The proportion of T cells in the infected group did not significantly change during infection (Figures 1H and I), but their number significantly decreased over time (Figure 1J).

These results highlight the dynamic changes in immune cells in the spleens of chickens following genotype VII NDV infection. Notably, there was a significant decrease in the number of SMNCs, an increase in the macrophage proportion, a decrease in both the B-cell proportion and the B-cell count, and a stable decrease in the T-cell proportion.

### Changes in T-cell subsets and splenocyte apoptosis following viral infection

**Figure 2:**
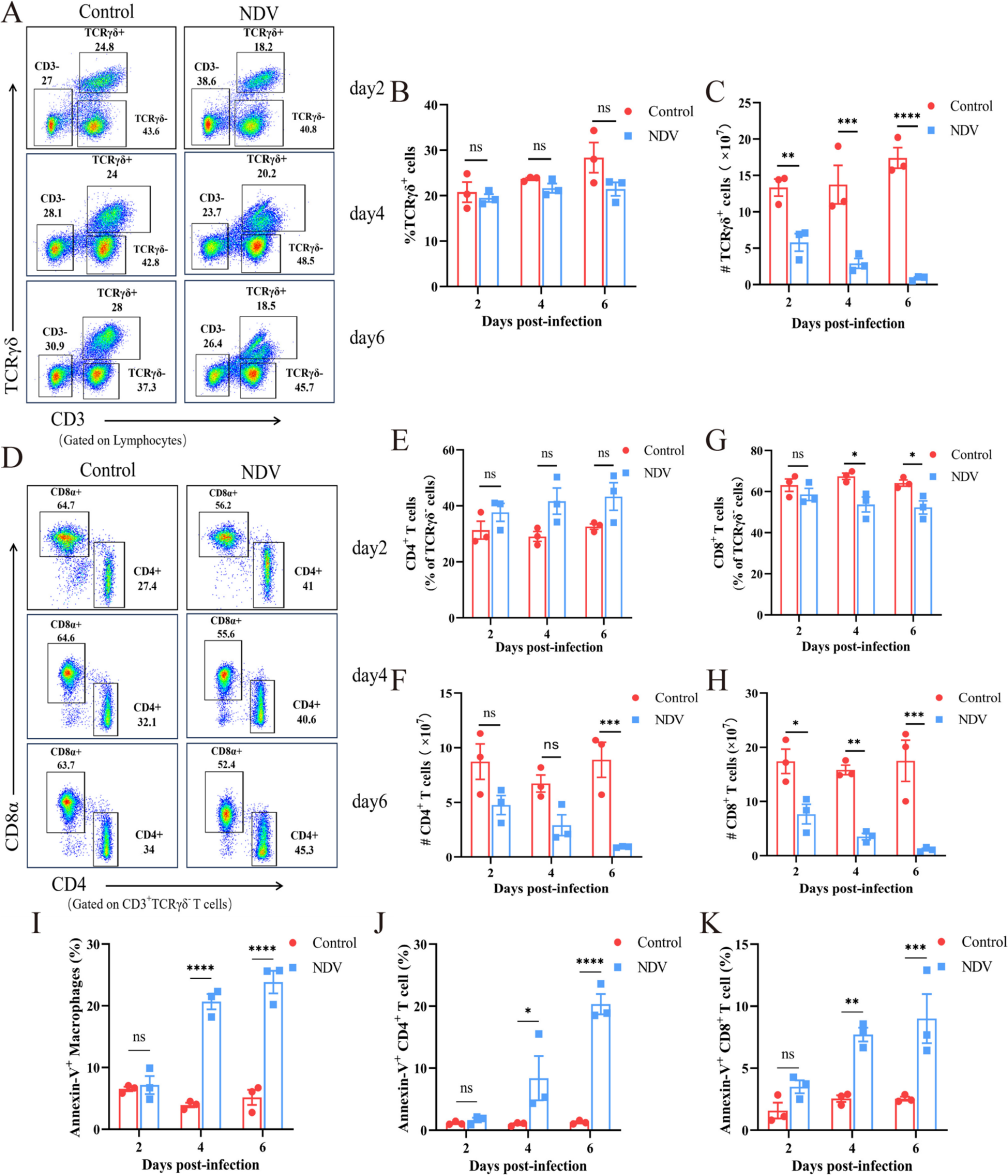
**Alterations in T-cell subsets and apoptotic cell proportions after viral infection. **(**A**) Representative dot plots showing changes in the numbers of TCRγδ^+^ T cells and CD3^+^ TCRγδ^−^ T cells. (**B**) Proportion of TCRγδ^+^ T cells among lymphocytes. (**C**) The absolute number of TCRγδ^+^ T cells in the spleen. (**D**) Representative dot plots showing changes in the proportions of CD4^+^ T cells and CD8^+^ T cells among CD3^+^ TCRγδ^−^ T cells. (**E**) Proportion of CD4^+^ T cells among CD3^+^ TCRγδ^−^ T cells. (**F**) The absolute number of CD4^+^ T cells in the spleen. (**G**) Proportion of CD8^+^ T cells among CD3^+^ TCRγδ^−^ T cells. (**H**) The absolute number of CD8^+^ T cells in the spleen. Proportion of apoptotic macrophages (**I**), CD4^+^ T cells (**J**), and CD8^+^ T cells (**K**) in the spleen.

To further investigate changes in various T-cell subsets, we conducted flow cytometric analysis of TCRγδ^+^ T cells, CD4^+^ T cells, and CD8^+^ T cells in the chicken spleen (Figures 2A and D). The results revealed that the percentage of TCRγδ^+^ T cells among lymphocytes in the infected group was not significantly different from that in the control group (Figure 2B). However, the absolute number of TCRγδ^+^ T cells was significantly lower in the infected group than in the control group and continued to decrease (Figure 2C). The percentage of CD4^+^ T cells among the TCRγδ^−^ cells did not significantly differ from that among the control group after infection (Figure 2E), whereas the percentage of CD8^+^ T cells among the TCRγδ^−^ cells significantly decreased at 4 and 6 dpi (Figure 2G). In terms of cell numbers, fewer CD4^+^ and CD8^+^ T cells were detected in the infected group than in the control group at 2 dpi, and these numbers continued to decrease (Figures 2F and H). Analysis of splenocyte apoptosis revealed no significant changes in the number of macrophages, CD4⁺ T cells, or CD8⁺ T cells at 2 dpi. However, significantly increased apoptosis rates were observed in these cells at 4 and 6 dpi compared with those in the control group (Figures 2I–K).

In summary, viral infection caused significant reductions in all splenic T lymphocyte subsets in chickens, accompanied by marked apoptosis of macrophages, CD4⁺ T cells, and CD8⁺ T cells at 4 and 6 hpi.

### NDV exhibits a marked tropism for macrophages and induces apoptosis in SMNCs

**Figure 3:**
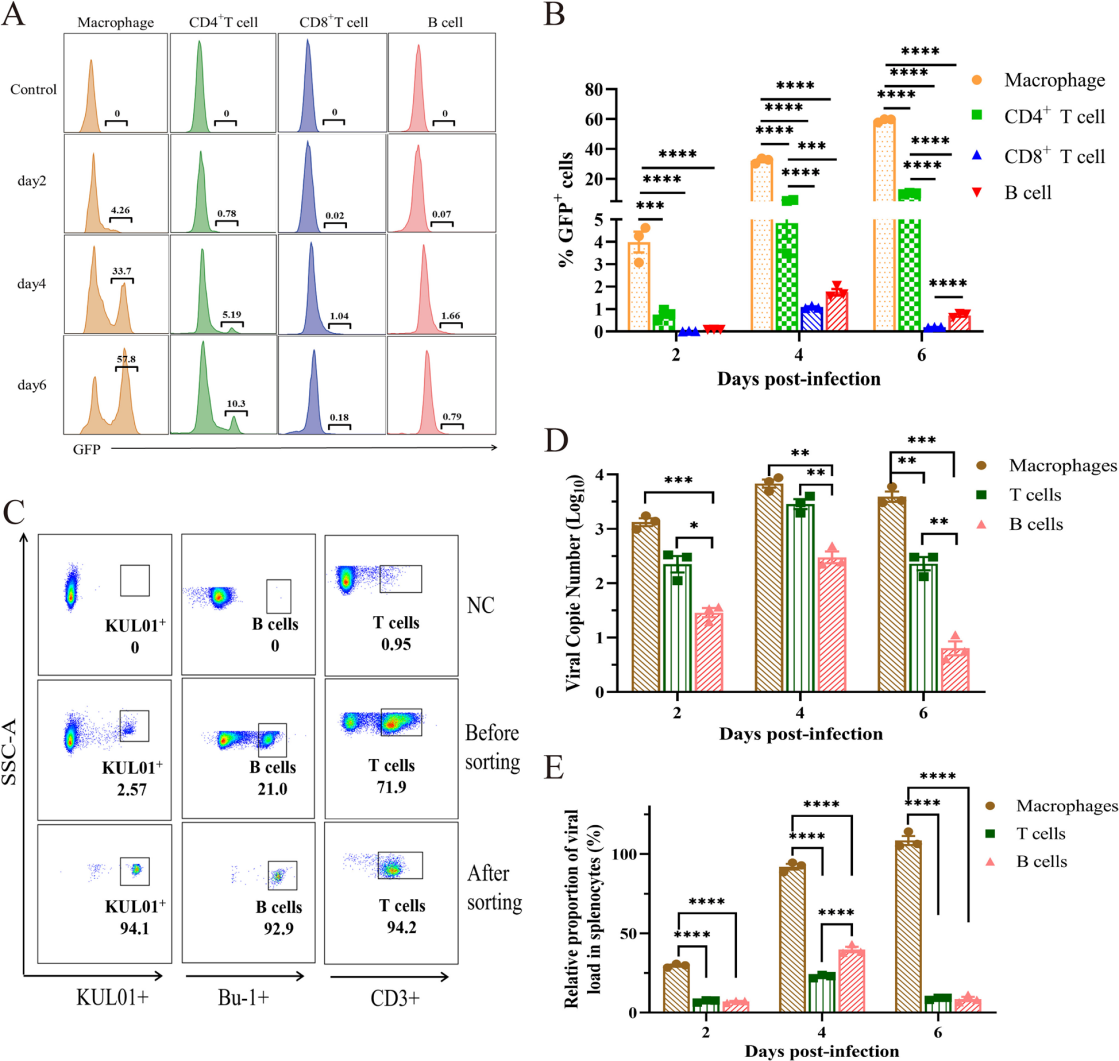
**NDV exhibits a marked tropism for macrophages. **(**A**) Representative peak plots showing the proportion of GFP^+^ cells in chicken SMNCs. (**B**) The proportion of GFP^+^ cells in chicken SMNCs. (**C**) High-purity B cells, T cells and KUL01^+^ cells were isolated from chicken SMNCs at 2, 4, and 6 dpi using FACS. (**D**) The viral load within these three types of cells was quantified using quantitative fluorescence detection. (**E**) The relative viral content of each cell was determined by calculating the ratio of the number of intracellular viral copies to the relative proportion of that cell. Statistical graphics were generated using GraphPad Prism software.

To assess the infectivity of various splenocyte types in chickens by NDV, we used flow cytometry to detect the expression of GFP, which serves as a marker for the virus, in different cell types. No green fluorescent signal from the virus was detected in the SMNCs of the control group (Figure 3A). In the immune cells from the spleens of infected chickens, the proportion of GFP^+^ cells in macrophages was the highest and was significantly higher than that in the other three immune cell types (Figure 3B). At 4 dpi, the proportion of GFP^+^ T cells among CD4^+^ T cells significantly increased, although it remained much lower than that among macrophages. We then used FACS to isolate macrophages, B cells, and T cells from SMNCs for viral load detection. The purity of the sorted cells exceeded 90%, as determined by FACS analysis (Figure 3C). We subsequently utilized qRT‒PCR to measure the viral load within the sorted T cells, B cells, and macrophages. The data revealed that the viral copy number in macrophages remained significantly higher than those in T cells and B cells at 2, 4 and 6 dpi (Figure 3D). To more accurately evaluate the viral tropism for macrophages, T cells, and B cells in the spleen, we determined the relative viral load by calculating the ratio of viral copies to the relative proportion of each cell type. A higher relative viral load indicated a greater susceptibility of the cell to NDV. The results demonstrated that the relative viral content of macrophages was significantly greater than that of the other two cell types (Figure 3E). These findings suggest that genotype VII NDV exhibits preferential tropism for splenic macrophages in chickens, identifying them as the primary target cells following viral infection of the spleen.

### Effects of NDV infection on T-cell death

**Figure 4:**
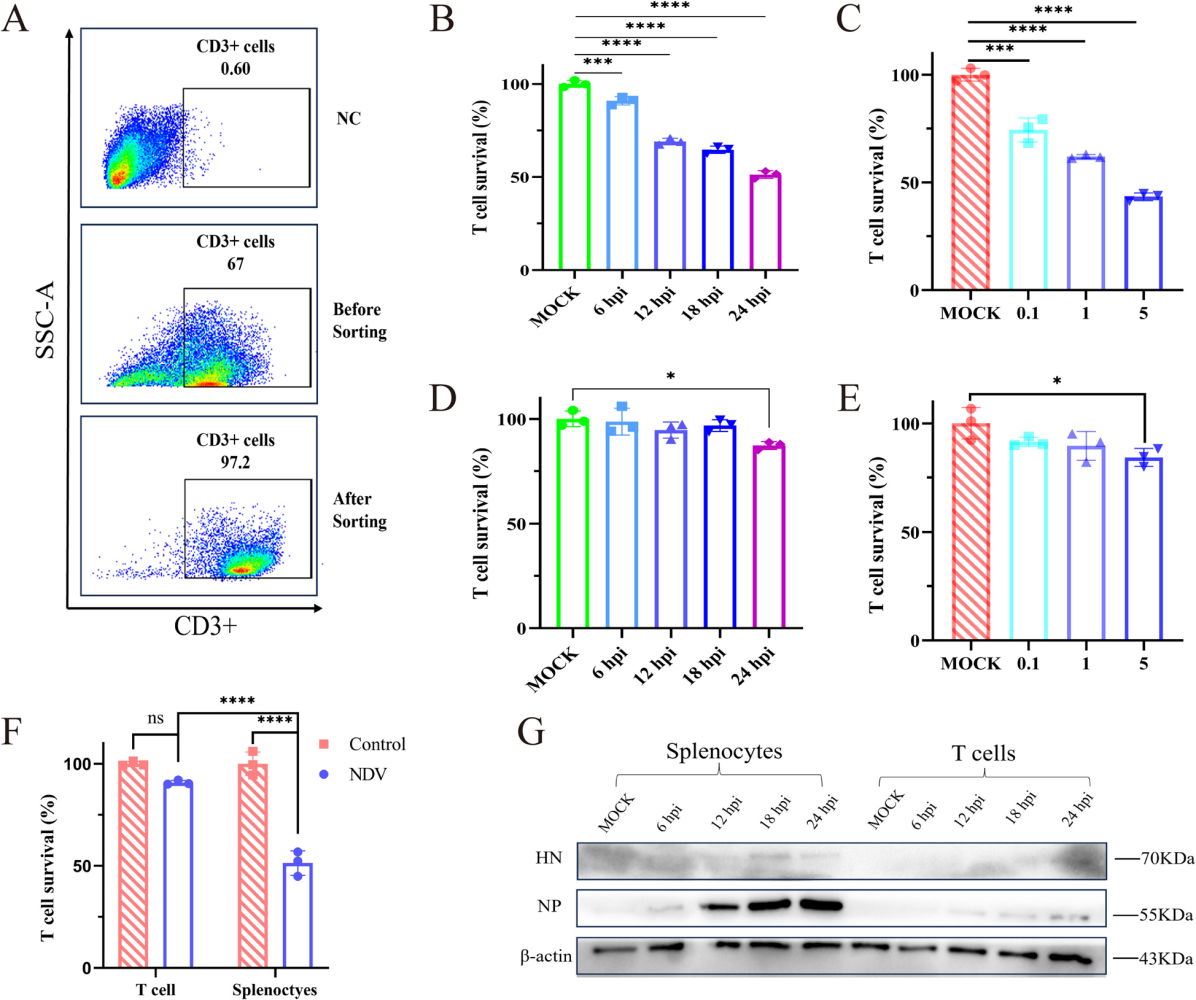
**Detection of T-cell death in NDV-infected SMNCs.** Chicken spleens were sterilely harvested and prepared in a single-cell suspension. T cells were then labelled with a biotin-conjugated anti-chicken CD3 antibody. (**A**) The cells were collected using streptavidin magnetic beads, and the purity of the T cells was assessed using flow cytometry. (**B**) T-cell survival in SMNCs was assessed at different time points following viral infection. (**C**) T-cell survival in SMNCs was assessed after infection with MOIs of 0.1, 1, and 5. (**D**) T-cell survival was assessed in isolated T cells at different time points following viral infection. (**E**) T-cell survival was evaluated in isolated T cells after infection with MOIs of 0.1, 1, and 5. (**F**) T-cell survival was compared between isolated T cells and SMNCs at 24 hpi with an MOI of 1. (**G**) Detection of the NP via western blotting. SMNCs and T cells were infected with NDV at an MOI of 1 for 6, 12, 18, or 24 hpi and then harvested and lysed in RIPA buffer containing PMSF. Western blotting was then performed using specific antibodies to detect the expression levels of NDV NP and HN.

T cells represent the greatest proportion of chicken splenic lymphocytes; however, our previous research demonstrated a significant decrease in T-cell numbers following NDV infection. To investigate the mechanisms underlying T-cell depletion, we utilized magnetic bead-based cell sorting to purify T cells from chicken spleens, resulting in a purity exceeding 95% after sorting (Figure 4A). SMNCs and sorted T cells were subsequently infected with rI4-EGFP, and T-cell viability was assessed using FACS. After viral infection, T-cell viability decreased over time (Figures 4B and D) and in a dose-dependent manner (Figures 4C and E) in both the splenocyte and the sorted T-cell groups. However, the decrease in T-cell viability was less severe in the sorted T-cell group than in the splenocyte group. Furthermore, SMNCs and sorted T cells were infected with rI4-EGFP at an MOI of 1, and T-cell viability was assessed at 24 hpi. We found that T-cell viability was significantly lower in the splenocyte population than in the sorted T-cell population (Figure 4F). Additionally, we detected viral protein expression in both cell groups, which exhibited a time-dependent pattern. The NP expression level was significantly higher in the splenocyte group than in the sorted T-cell group (Figure 4G). These findings indicate that when SMNCs are infected with NDV in vitro, substantial T-cell death is induced, with a more severe degree of cell death than in the purified T-cell group infected with NDV.

### Macrophages enhance the infectivity of NDV in T cells

**Figure 5:**
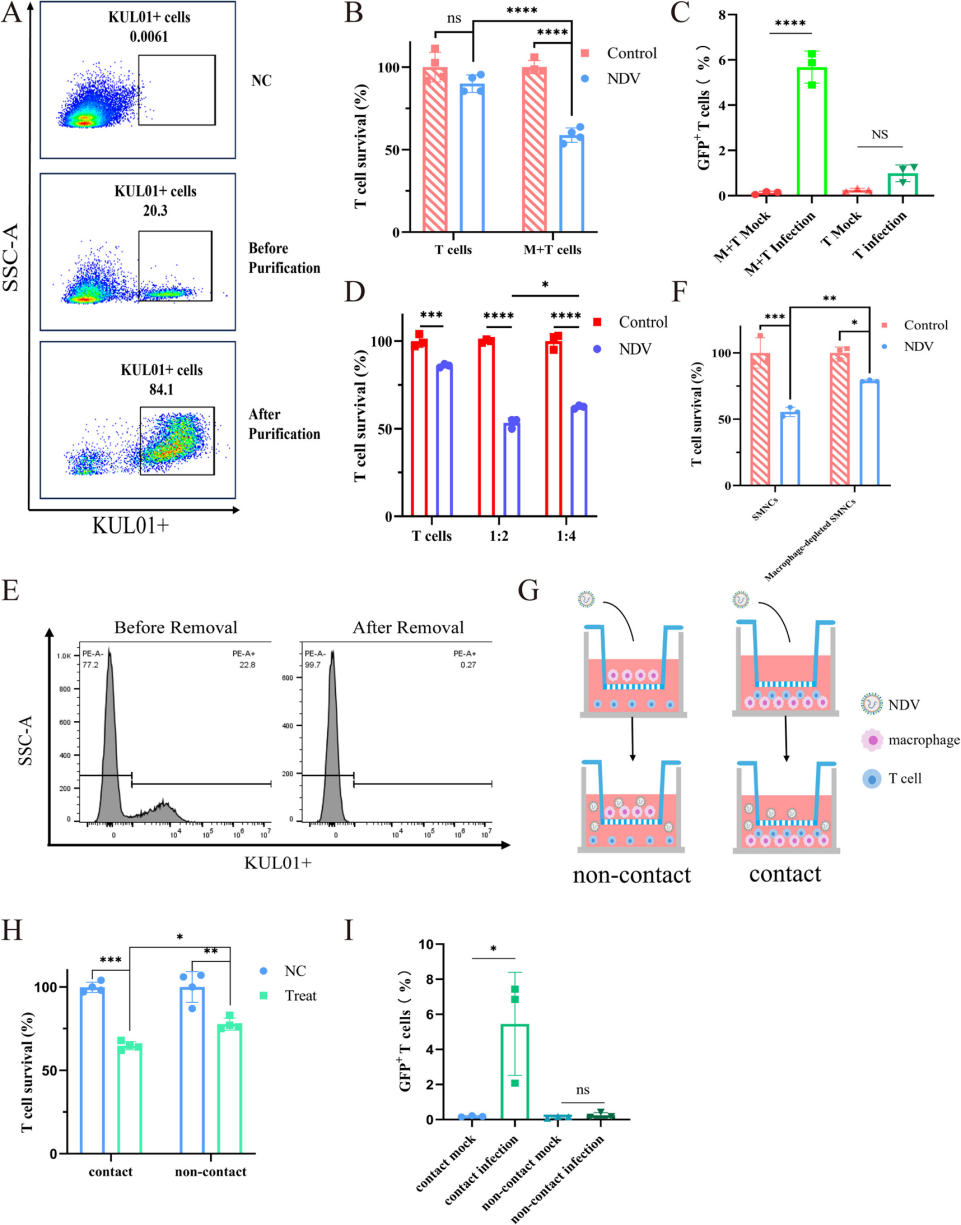
**The role of macrophages in T-cell death.** SMNCs were cultured in a 10 cm dish, and the supernatant was discarded, followed by washing with PBS twice every 24 h. The adherent cells at the bottom were collected, and their purity was determined by flow cytometry following incubation with macrophage surface marker antibodies at 48 hpi (**A**). Macrophages and T cells were cocultured at a ratio of 1:4, and both cocultured cells and T cells were infected with rI4-EGFP at an MOI of 1. After 24 h, the samples were collected, and T-cell viability (**B**) and the proportion of CD3^+^GFP^+^ cells (**C**) were assessed by flow cytometry. The macrophages and T cells were cocultured at ratios of 1:2 and 1:4, and both the cocultured cells and the T cells were infected with rI4-EGFP at an MOI of 1. After 24 h, the samples were collected, and T-cell viability was assessed by flow cytometry (**D**). The macrophages were removed from the SMNCs using the adherence method (**E**). SMNCs with and without macrophages were subsequently infected with the virus at an MOI of 1, and T-cell viability was determined by flow cytometry (**F**). (**G**) Schematic diagram of the coculture model in which macrophages (upper chamber) and T cells (lower chamber) were separated using a 0.4-μm-pore Transwell insert. Following infection with NDV at an MOI of 1, T-cell viability (**H**) and the proportion of EGFP^+^ T cells (**I**) were assessed at 24 hpi in both the contact and noncontact coculture groups.

Macrophages were identified as the primary target cells for NDV infection in chicken spleens, and we hypothesized that macrophages might play an important role in T-cell death. To investigate this hypothesis, macrophages were isolated with a purity of 80–90% using a two-step adherence method (Figure 5A) and cocultured with the sorted T cells. After the coculture model was infected with rI4-EGFP, the survival rate of T cells in the coculture group was significantly lower than that in the sorted T-cell group (Figure 5B), and the proportion of NDV-positive T cells was higher in the coculture group (Figure 5C). Flow cytometric analysis also revealed no significant increase in the EGFP⁺ T-cell proportion compared with that in the control group (Figure 5C), confirming the low infection efficiency of NDV in T cells. We also found that a higher proportion of macrophages led to a more pronounced decrease in T-cell survival (Figure 5D). Furthermore, we removed macrophages from SMNCs, reducing the macrophage proportion from 22.8 to 0.27% (Figure 5E). Upon elimination of macrophages, the T-cell survival rate increased significantly (Figure 5F). Following NDV infection, macrophages induced T-cell death without direct contact when separated by a 0.4-μm-pore Transwell system, although this effect was less pronounced than that with direct contact (Figures 5G and H). Furthermore, T-cell infection rates were significantly higher in contact cultures than in noncontact cultures (Figure 5I).

These data indicate that macrophage-derived cytokines/mediators induce T-cell death, whereas direct cell contact enhances NDV infection in T cells.

### Macrophages induce T-cell apoptosis after NDV infection

**Figure 6:**
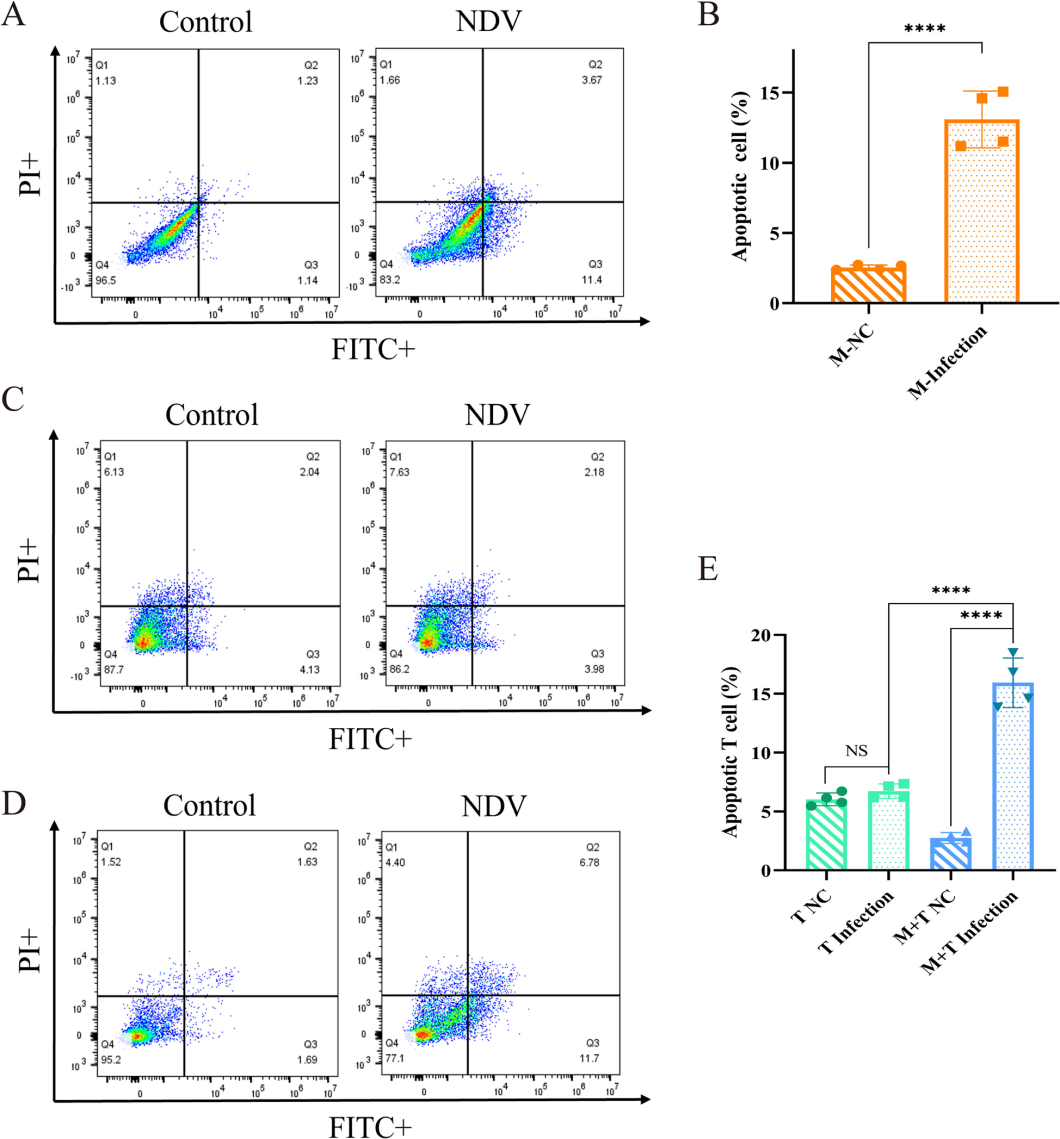
**Severe apoptosis of T cells among cocultured cells.** Cells were infected with rI4-EGFP at an MOI of 1, and cell samples were collected at 24 hpi. The cells were incubated with Annexin V-FITC apoptosis detection reagent and an anti-chicken CD3 antibody. The apoptosis levels of primary macrophages (**A**), T cells (**C**), and T cells in coculture (**D**) were assessed via flow cytometry. (**B, E**) Statistical analysis of the results was performed using GraphPad Prism 9.0 software.

Macrophages can induce T-cell apoptosis through the extrinsic apoptosis pathway [29]. To evaluate T-cell apoptosis in the T-cell–macrophage coculture model, we first labelled T cells with a CD3-SPRD antibody and then used an Annexin V-FITC/PI apoptosis detection kit. The results indicated that rI4-EGFP infection led to significant apoptosis in splenic macrophages (Figures 6A and B), which is consistent with the findings of previous reports. Infection of the sorted T cells with the virus did not induce apoptosis (Figures 6C and E). However, significant T-cell apoptosis was observed in the coculture model (Figures 6D and E). These findings indicate that NDV infection does not directly induce T-cell apoptosis, and the presence of macrophages may explain this phenomenon.

## Discussion

NDV causes severe damage to lymphoid tissues [30, 31]. Hu et al. reported that, in addition to genotype VII NDV strains, the genotype IX F48E8 and genotype IV Herts/33 strains also induced lymphocyte depletion and necrosis in the chicken spleen [32]. However, the extent of lymphocyte depletion caused by these two strains was significantly lower than that induced by the genotype VII strains. These findings suggest that along with genotype VII strains, other NDV genotypes may also induce T-cell death in the chicken spleen, although the pathogenicity of these strains is weaker. While prior studies have documented macroscopic pathological changes in the spleen post-NDV infection, they have focused primarily on broad fluctuations in splenic cell populations, leaving detailed analyses of individual immune cell subsets unexplored. To address this gap, we employed multicolour flow cytometry with a panel of fluorochrome-conjugated antibodies to analyse dynamic changes in splenic macrophages, T cells, and B cells at various post-infection time points. Our findings revealed a significant decrease in the numbers of SMNCs, T cells, and B cells during NDV infection. Notably, macrophage numbers increased at 4 dpi but returned to levels comparable to those of the control group at 6 dpi, likely due to the drastic reduction in SMNCs. Analysis of apoptosis revealed that macrophages, CD4⁺ T cells, and CD8⁺ T cells underwent apoptosis following NDV infection. Although CD4⁺ T cells constituted a minor population in the chicken spleen, they exhibited more severe apoptosis than the more abundant CD8⁺ T cells did. Moreover, the proportion of EGFP⁺CD4⁺ T cells was significantly higher than that of EGFP⁺CD8⁺ T cells, indicating that NDV has stronger infectivity and cytopathic effects on CD4⁺ T cells than on CD8⁺ T cells. High levels of apoptosis were detected in the macrophages, but their numbers increased in the infected spleens at 4 dpi. This increase may be attributed to the recruitment of monocytes from the bloodstream to replace the dying macrophages. Furthermore, higher levels of viral replication were detected in macrophages, identifying them as the primary target for viral infection. Previous research has shown that T-cell-deficient mice exhibit significantly higher mortality rates and inflammatory cytokine levels after viral infection than wild-type mice do, suggesting that T-cell-mediated suppression of innate immune responses may be critically important in acute infection pathogenesis [33]. Given that T cells make up approximately 60% of splenic lymphocytes in chickens and play a pivotal role in NDV defence [27], a reduction in this subset could undermine cellular immunity and facilitate genotype VII NDV infection. Although NDV infection causes splenic T-cell depletion, vaccination against NDV significantly enhances the expansion of T cells in the peripheral blood circulation of chickens, thereby eliciting protective immune responses [34]. This study provides a comprehensive profile of dynamic splenocyte variation following genotype VII NDV infection and offers crucial insights into viral pathogenesis.

To elucidate the relationship between T-cell reduction and viral infection, we attempted to isolate chicken splenic T cells. While methods for mammalian T-cell isolation are well established [29, 35], protocols for avian T cells require refinement. In this study, both flow cytometry and magnetic bead sorting were employed to enrich chicken splenic T cells. We found that magnetic bead sorting could successfully increase T-cell yield and maintain viability, providing a solid foundation for subsequent experiments. The data revealed that, following NDV infection, T-cell mortality was significantly higher in the mixed splenic cell population than in the sorted T-cell population. Additionally, western blot analysis revealed relatively low NDV viral protein expression in the sorted T cells. These findings suggest that genotype VII NDV has a limited ability to infect T cells directly and is not solely responsible for T-cell depletion.

Macrophages are the primary target cells of genotype VII NDV, and their numbers increase significantly during infection. To clarify the role of macrophages in T-cell reduction during genotype VII NDV infection, we prepared high-purity macrophages using a double adhesion culture method and cocultured them with T cells to create a coculture model. After viral infection, severe T-cell death occurred in the coculture group, accompanied by a marked increase in the number of virus-infected T cells. Conversely, removing macrophages from SMNCs significantly increased T-cell survival, confirming the crucial role of macrophages in promoting T-cell death and viral infection. Transwell experiments indicated that macrophage-secreted factors independently induce T-cell apoptosis. However, direct contact between macrophages and T cells significantly increased both the level of viral infection in T cells and their death rate. Since NDV can cause cell death, this effect may result from the combined action of the virus and macrophage-derived factors. However, the underlying mechanisms require further investigation.

NDV infection can induce various modes of cell death, including apoptosis [36], necroptosis [37], ferroptosis [38], and pyroptosis [39]. Apoptosis is recognized as the primary mechanism through which NDV infection induces cell death and mediates pathogenicity [31]. In the later stages of infection, both the intrinsic and extrinsic apoptotic pathways become activated. Previous studies have indicated that NDV can induce apoptosis in SMNCs [10]. Our results showed that direct infection of T cells by genotype VII NDV did not cause significant apoptosis. However, when macrophages were cocultured with T cells, marked T-cell apoptosis occurred. Therefore, macrophages promote T-cell apoptosis during NDV infection. A previous study revealed that macrophages highly expressed FasL on their surface, which could interact with FAS on T cells to induce T-cell death [29]. Since the Fas–FasL signalling pathway can activate the apoptotic pathway, we also examined macrophage apoptosis of macrophages and found that NDV infection caused macrophage apoptosis. Thus, we hypothesize that the Fas‒FasL signalling pathway may play a critical role in macrophage-mediated T-cell apoptosis.

In summary, genotype VII NDV triggers a potent immune response through a complex network of immune cell alterations and regulatory mechanisms. Macrophages not only serve as direct viral targets but also contribute to the infection and damage of other immune-related cells, thereby enhancing viral pathogenicity. These findings improve our understanding of the pathogenic mechanisms of genotype VII NDV and offer a theoretical basis for the development of future antiviral strategies.

## Supplementary Information

**Additional file 1. Gating strategies of panel 1 to identify chicken B cells and myeloid lineages.** Splenic mononuclear cells were harvested from 4-week-old chickens and surface stained with antibody cocktails. The leukocytes were gated as CD45 positive (**A**), and single cells were subsequently gated using FSC-A and FSC-H (**B**). Lymphocyte populations were subsequently gated using FSC-A/SSC-A parameters (**C**), and Bu-1+ B cells were defined (**F**). By excluding Bu-1+B cells (**E**), KUL01+ cells were identified (**D**).

**Additional file 2. Gating strategies of panel 2 and panel 3 to identify T-cell subsets.** Lymphocytes were initially gated using FSC-A versus SSC-A (**A**), with single cells confirmed by FSC-A and FSC-H (**B**). T cells were identified as CD3 positive (**C, D**). Live cells were defined as FVD eFluor 780-negative cells (G). Subsequent analysis of CD3 and TCRγδ expression revealed CD3^+^TCRγδ^+^ (γδ T cells), CD3^+^TCRγδ^−^, and CD3^−^TCRγδ^−^ populations (**F**). CD3^+^TCRγδ^−^ T cells were subdivided into TCRγδ^−^CD3^+^CD4^+^ and TCRγδ^−^CD3^+^CD8α^+^ subsets (**E**).

## Data Availability

All the data generated or analysed during this study are included in this published article.
