# Supplementary figures and images for: Macrophages are the target cells of genotype VII Newcastle disease virus and promote the infection and apoptosis of chicken splenic T cells
Source: Vet Res. 2025 Oct 30;56:207. doi: 10.1186/s13567-025-01631-8 (PMC12577440; doi:10.1186/s13567-025-01631-8)

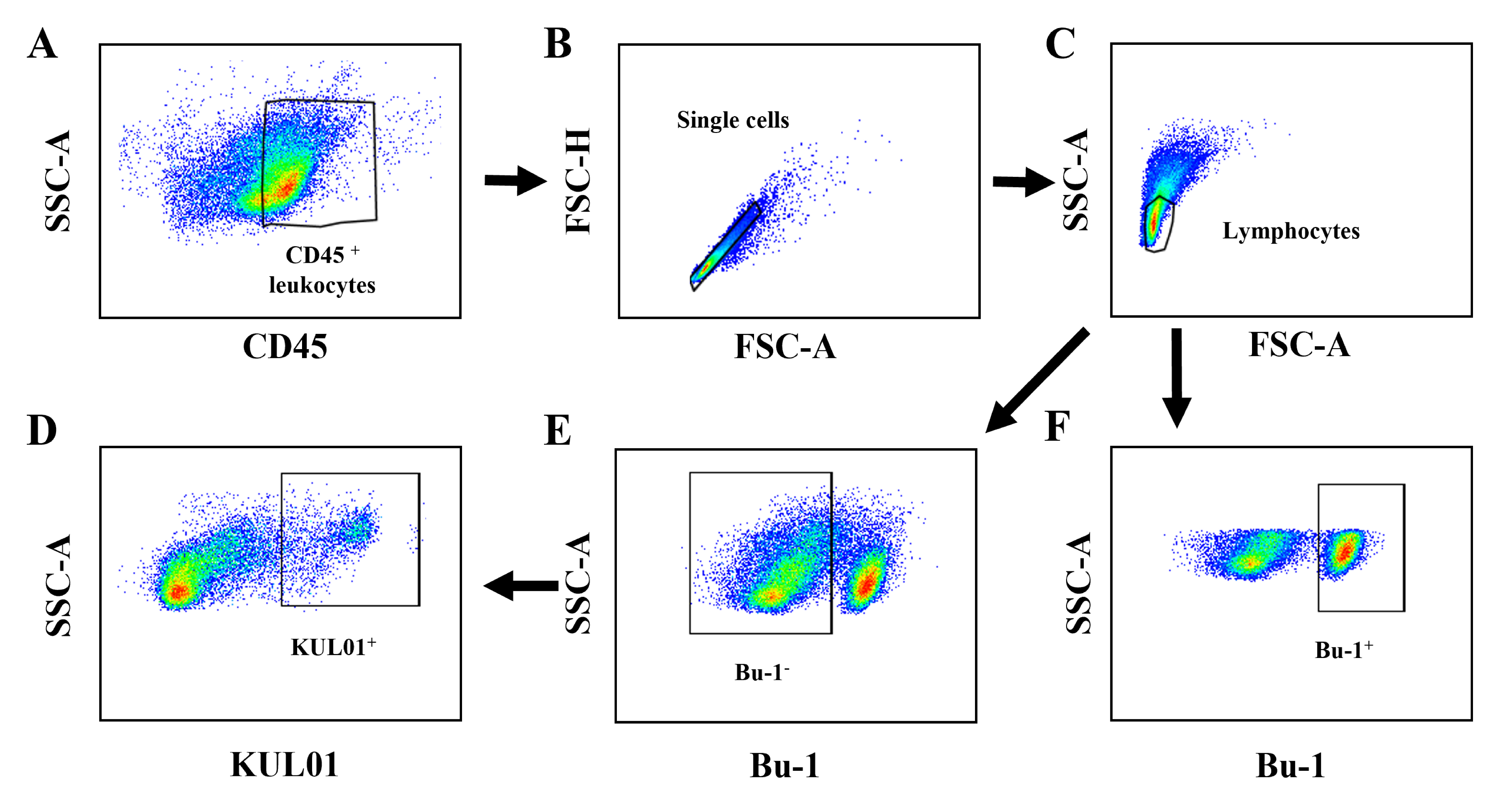

Supplement: Supplementary file 1 — Additional file 1. Gating strategies of panel 1 to identify chicken B cells and myeloid lineages. Splenic mononuclear cells were harvested from 4-week-old chickens and surface stained with antibody cocktails. The leukocytes were gated as CD45 positive (A), and single cells were subsequently gated using FSC-A and FSC-H (B). Lymphocyte populations were subsequently gated using FSC-A/SSC-A parameters (C), and Bu-1+ B cells were defined (F). By excluding Bu-1+B cells (E), KUL01+ cells were identified (D). [file 13567_2025_1631_MOESM1_ESM.tif]

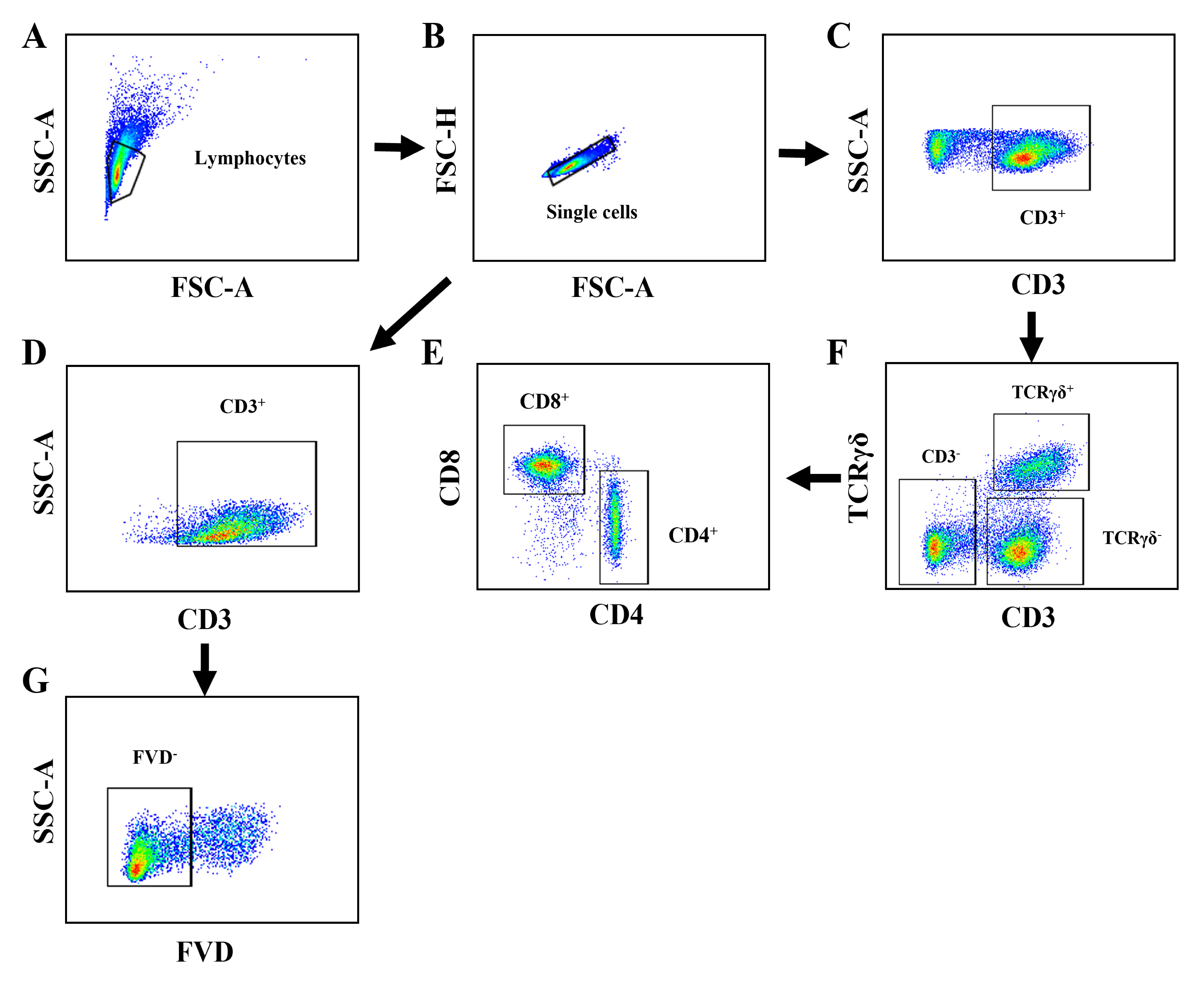

Supplement: Supplementary file 2 — Additional file 2. Gating strategies of panel 2 and panel 3 to identify T-cell subsets. Lymphocytes were initially gated using FSC-A versus SSC-A (A), with single cells confirmed by FSC-A and FSC-H (B). T cells were identified as CD3 positive (C, D). Live cells were defined as FVD eFluor 780-negative cells (G). Subsequent analysis of CD3 and TCRγδ expression revealed CD3+TCRγδ+ (γδ T cells), CD3+TCRγδ−, and CD3−TCRγδ− populations (F). CD3+TCRγδ− T cells were subdivided into TCRγδ−CD3+CD4+ and TCRγδ−CD3+CD8α+ subsets (E). [file 13567_2025_1631_MOESM2_ESM.tif]
